# Management of acute colonic pseudo-obstruction: opportunities to improve care?

**DOI:** 10.1308/rcsann.2024.0017

**Published:** 2024-03-06

**Authors:** Z Khan, CP Challand, MJ Lee

**Affiliations:** ^1^ University of Sheffield, UK; ^2^ Sheffield Teaching Hospitals, UK

**Keywords:** Pseudo-obstructions, Outcomes

## Abstract

**Background:**

Acute colonic pseudo-obstruction (ACPO) is a functional bowel obstruction characterised by colonic dilatation in the absence of mechanical obstruction on imaging. Complications include bowel ischaemia, perforation and death. The aim of this study was to explore outcomes for patients treated for ACPO and to assess adherence to current ACPO treatment guidelines.

**Methods:**

This is a retrospective service evaluation and included patients with a diagnosis of ACPO between 1 March 2018 and 31 March 2023. Process measures were identified following discussion with the clinical team from published guidance. Patients were identified using clinical coding and radiological text reports. Cases were eligible for inclusion if they had radiologically confirmed ACPO. Data were collected following review of patient notes into Microsoft Excel. Descriptive analysis was performed with no formal statistical assessment.

**Results:**

A total of 45 patients were identified, of whom 13 were admitted under general surgery. All patients received admission bloods (*n*=45). Nearly all patients had computed tomography imaging (43/45, 96%). Only 3/45 (6.7%) of the patients received optimal conservative management (intravenous infusion, nil by mouth, flatus tube, treatment of reversible causes). In all, 11/45 (24%) required further treatment, of whom 7 received this within 72 h. The leading (11/45) complication following diagnosis of ACPO was hospital-acquired pneumonia. Mortality was seen in 9/45.

**Conclusions:**

ACPO is often managed remotely by general surgeons. This may impact on the quality of conservative management, and timeliness of endoscopic or pharmacological intervention. Further work is needed to optimise management.

## Background

Acute colonic obstruction is a surgical emergency, and can occur due to mechanical or nonmechanical pathologies.^1^ Acute colonic pseudo-obstruction (ACPO) is one such cause of nonmechanical bowel obstruction, with distension of the colon throughout its length to the anal sphincter without luminal pathology causing obstruction. The aetiology is not fully understood, but thought to result from dysregulation of the autonomic nervous supply of the bowel.^2–4^

The incidence of ACPO is currently estimated to be 100 cases in 100,000 for inpatient stays per year.^5^ Typically affected patients tend to be male, elderly, postoperative (particularly spinal trauma patients) and comorbid.^6–8^ Diagnosis is usually made from clinical history, an air-filled rectum on digital examination, and radiological evidence of colonic dilation with no evidence of mechanical obstruction.^3,6,7,9^ Complications include bowel ischaemia, perforation and death.

Current guidelines produced by the American Society of Colon and Rectal Surgeons (ASCRS) and the American Society for Gastrointestinal Endoscopy (SAGE) recommend full assessment and initial supportive management comprising keeping the patient nil by mouth (NBM), intravenous infusion (IVI), electrolyte replacement, avoidance of predisposing medications (anticholinergics and opiates), treatment of ongoing infections and flatus tube insertion.^1,10^ If symptoms persist for longer than 48–72h of supportive management, medical treatment with neostigmine and/or endoscopic colonic decompression is recommended due to risk of bowel ischaemia and perforation, which may result in further morbidity and mortality.^1,5^

The aim of this study was to explore adherence to guidelines in our practice.

## Methods

### Study design

This retrospective service evaluation explored outcomes of patients treated for ACPO at our institution, and assessed adherence to guidelines. Ethical approval is not required for this type of study; however, we secured approval from our trust Clinical Effectiveness Unit (CEU) before commencement (CEU registration 11372). The study is reported with reference to STROBE guidelines (Supplement 1).^11^

### Setting

This service evaluation was conducted retrospectively at a tertiary centre and included patients with a diagnosis of ACPO between 1 March 2018 and 31 March 2023.

### Sample identification

Patients were identified with the support of clinical coding using the codes ‘paralytic ileus’ from the ICD-10 manual (K56.0).^12^ In addition, text reports from radiology were searched for the following terms: ‘pseudo obstruction/pseudo-obstruction/pseudoobstruction’. Cases were eligible for inclusion if they had radiologically confirmed ACPO.

### Definitions

The following definitions were used:

• No standardised definition of ACPO exists.^13^ We defined ACPO as colonic dilatation in the absence of mechanical obstruction as demonstrated on computed tomography (CT) scan.
• Resolution was defined as the opening of bowels documented in patient notes. Definitions of complications such as hospital-acquired pneumonia or urinary tract infection were based on contemporaneous clinical notes. Adequate decompression was determined by the resolution of symptoms as assessed by the treating team (resolution of distension and bowels opening).
• Optimal conservative management was defined as keeping the patient NBM, administration of IVIs, initial decompression by flatus tube insertion via rigid sigmoidoscope, cessation of anticholinergic medications and opioids, and, if indicated, treatment of infection and correction of electrolyte abnormalities. This information was sought from patient notes, drug cards, handover documents and discharge summaries. Where elements could not be identified specifically, they were classified as not present in the data.
• Additional treatment was defined by the need for further treatment following conservative management. This comprised medical management with neostigmine, endoscopic decompression or surgery.

### Key pathway metrics

In discussion with the clinical team, we identified process measures from published guidance that were thought to be indicative of overall process quality.^1,10^ These are presented in Table 1.

**Table 1:** Standards generated based on current guidelines

Standard	How compliance to the standard was measured	Source document
Bloods on admission (100%)	Review of laboratory blood results on institution computer systems	ASCRS^10^
CT imaging to confirm diagnosis (100%)	Review of radiological scans and reports	ASCRS^10^
Optimal conservative management (IVI, NBM, flatus tube, cessation of contributing medications, electrolyte replacement if applicable, and treatment of infections if applicable) (80%)	Review of patient paper and electronic notes, drug charts and handover documents and discharge summaries	SAGE and ASCRS^1,10^
Additional treatment (neostigmine, endoscopy, surgery) within 72h of presentation in all patients in whom conservative management failed (80%)	Review of patient paper and electronic notes, drug charts, handover documents, procedure notes and discharge summaries	SAGE and ASCRS^1,10^

ASCRS = American Society of Colon and Rectal Surgeons; CT, computed tomography; IVI, intravenous infusion; NBM, nil by mouth; SAGE = American Society for Gastrointestinal Endoscopy.

### Data extraction and analysis

Patient notes were requested from medical records and online electronic notes systems were also viewed. Data were collected and input following review of patient notes in a data collection tool on Microsoft Excel. The data were coded and subsequently analysed in Microsoft Excel. All data were anonymised. Given the small sample size, no formal statistical analysis has been performed. Descriptive numbers only are presented.

## Results

Searches identified 344 patients: 175 through clinical coding and 169 from radiological scan reports. A total of 110 duplicates were found and removed to create a sample size of 234. Of these 234 patients, radiological scans were reviewed to confirm a diagnosis of ACPO, and this was confirmed in 48 patients. Notes were unavailable for 3 patients; thus, 45 patients were included in this study.

**Figure 1:**
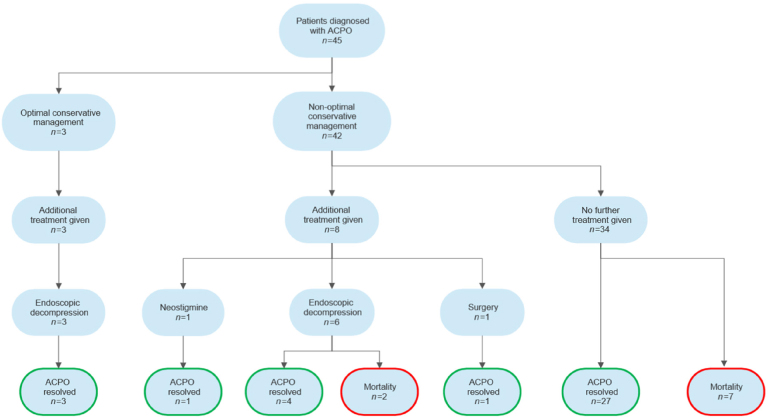
Process flow chart ACPO = acute colonic pseudo-obstruction.

Figure 1 demonstrates a process flow chart detailing optimal conservative management, further treatment and patient outcomes.

### Patient demographics

Patients were typically male and nonsmokers. Median age was 71 years old and the median body mass index was 25.6 (Table 2).

**Table 2:** Patient demographics

Characteristic	*N*=45*
Age at admission (years)	71 (58, 80)
Sex	
Female	17 (38%)
Male	28 (62%)
BMI (kg/m^2^)	25.6 (23.0, 28.0)
Unknown	3
MUST	
High risk	10 (22%)
Low risk	22 (49%)
Medium risk	8 (18%)
NR	5 (11%)
Smoking status	
Nonsmoker	37 (82%)
Not recorded	2 (4.4%)
Smoker	6 (13%)

*Median (IQR); *n* (%).

BMI = body mass index; MUST, Malnutrition Universal Screening Tool; NR, not reported.

Reason for admission fell under six broad categories: acute surgical presentation (abdominal pain, distension, constipation, change in bowel habit) (*n*=13), infection (*n*=8), electrolyte imbalance (*n*=3), postoperative patients following elective and emergency surgery for trauma (*n*=11), neurological disorder (*n*=5) and multifactorial (more than one of the aforementioned reasons) (*n*=5). Of the 45 patients, 13 (29%) were admitted with an acute surgical presentation. The remaining two-thirds of patients did not present directly to surgeons.

### Resolution of ACPO

Of the patients included in this study, ACPO resolved in 36/45 (80%) patients. All of those who did not resolve died (9/45, 20%).

**Figure 2:**
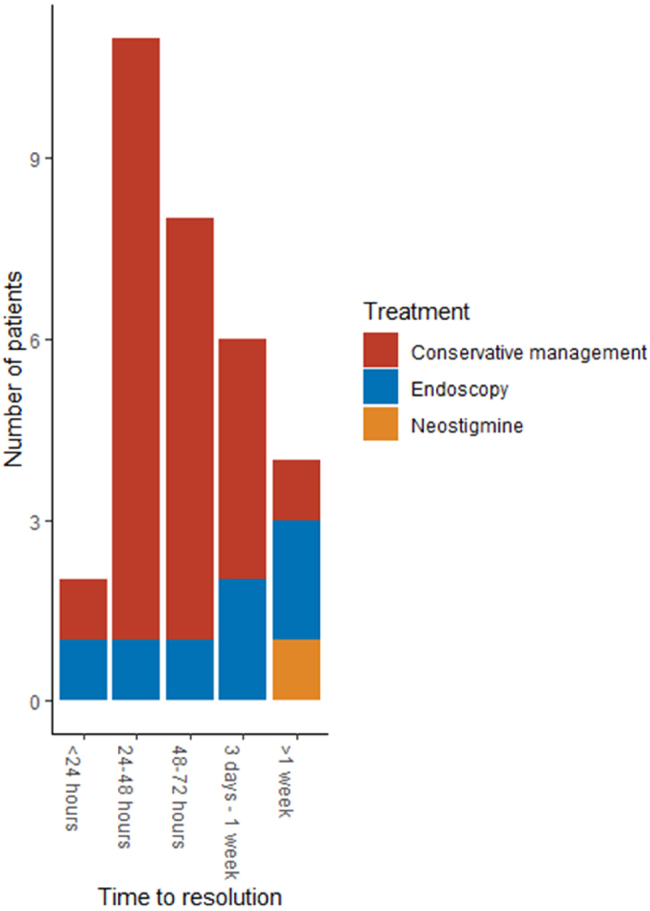
Graph showing time from diagnosis of ACPO to bowels opening signifying resolution of ACPO by treatment type. For two patients, bowel motions were not documented but the patients were ACPO resolved according to their discharge letters. ACPO = acute colonic pseudo-obstruction.

Of the patients who did resolve, 5/36 (14%) did not have the date of their bowel motions documented but were reported to be ACPO resolved on discharge letters. Of the 31/36 (86%) patients who did have their bowel motions recorded, the range of resolution time from diagnosis was 0–14 days with a median of 3 days. Figure 2 demonstrates the time for resolution of ACPO by maximal treatment received, documented here as time from ACPO diagnosis to bowels opening. The majority of patients with ACPO resolved by 24–72h and received conservative management and endoscopic decompression.

### Complications

The most common complications observed in the cohort were mortality (*n*=9) and hospital-acquired pneumonia (*n*=11). This was followed by an unplanned critical care (CC) admission (*n*=5), ischaemia/perforation (*n*=3), urinary tract infection (*n*=3), deep vein thrombosis/pulmonary embolism (*n*=2) and other (*n*=2). Other includes one patient who went on to develop a mechanical obstruction and another patient who was found to have a retroperitoneal tumour.

### Use of imaging

The majority of patients (43/45) were assessed with a CT-abdomen pelvis. The two patients who did not undergo CT did have a plain abdominal x-ray assessment of the abdomen that suggested pseudo-obstruction as the diagnosis. No reason was cited in the notes for why a CT scan was not performed.

### Use of optimal conservative management

**Figure 3:**
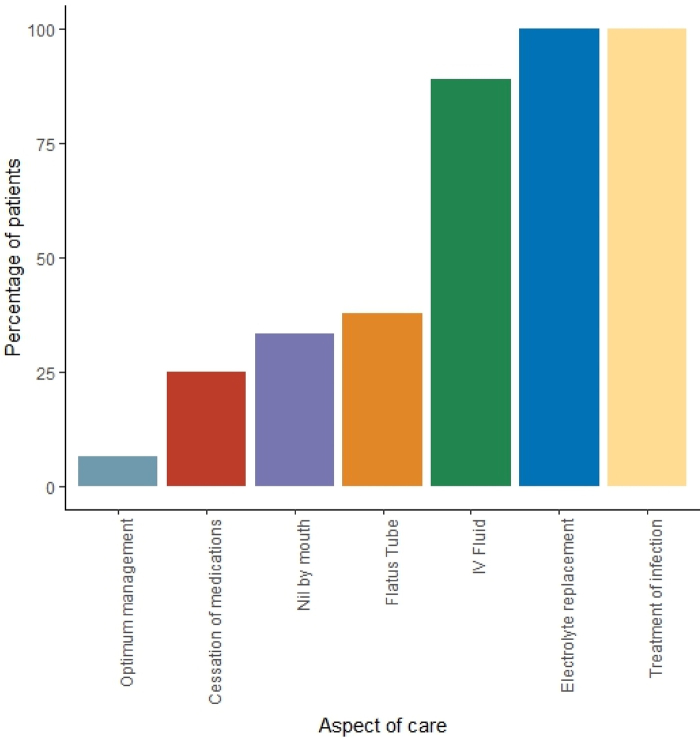
Conservative management. A graph demonstrating the proportion of patients receiving optimal conservative management broken down by treatment modality.

The majority of patients (42/45, 93%) did not receive optimal conservative management, with only 3/45 (6.7%) of patients receiving full conservative management. All patients that required correction of electrolyte imbalances (23/23) and treatment of infection (17/17), received this. Figure 3 highlights compliance with conservative management broken down by treatment modality. Only one-third of patients were kept NBM and only a quarter of the patients had their anticholinergic medications and opiate medications withheld. Less than 40% of patients received flatus tube decompression.

### Additional treatment (neostigmine, endoscopy, surgery) within 72h of presentation if indicated

One patient received pharmacological management with neostigmine, and this treatment was given within 72h of diagnosis with subsequent resolution. One patient went on to have surgical intervention at >72h after diagnosis. This patient did not receive optimum conservative management, pharmacological management, or endoscopic decompression. Nine patients underwent endoscopic decompression. Of the nine patients, only six received timely management in <72h.

## Discussion

This study reports on processes and outcomes in the management of ACPO in a single institution over several years. It highlights some challenges in management that will likely be relevant to other institutions. Although mortality was not the focus of the study, and we lack in-depth data on mortality, the reported mortality rate of 20% (9/45) is clinically significant. This is concordant with the literature, and is a persistent figure over time.^14^ Notably, the greatest mortality is seen in this group when surgery is required. This may reflect a failure of conservative management or a failure to diagnose and act on the condition in a timely manner. Our findings suggest that ischaemia or perforation occurred in 6.7% (3/45) of patients, and, of these patients, the mortality rate was 67% (2/3).

This report suggests that some aspects of management are well performed, notably the performance of baseline blood tests and cross-sectional imaging with CT scans to confirm diagnosis. However, in common with other conditions, there remains a persistent use of plain abdominal radiography, which provides limited information.^15^ Easily reversible causes of ACPO, such as electrolyte imbalance and management of infection, were also treated. None of these actions are specific to ACPO. It is reasonable to expect patients with new onset abdominal pain to have baseline blood tests and CT scan to aid diagnosis. It is also not unreasonable to expect actions to correct electrolyte abnormalities on these bloods, or to initiate treatment of an infection.

As management moves into disease-specific aspects of complexity, the completion rates of relevant interventions declines. This is evidenced by only 6.7% (3/45) patients receiving optimal conservative management. The main areas to improve would be to ensure patients are kept NBM, contributing medications are held and flatus tube decompression is attempted. Additionally, 36.3% (4/11) of patients who required additional management beyond conservative management received this >72h from presentation, highlighting an area for improvement to ensure that all patients receive timely further management.

With such opportunities to improve care, we must consider systemic reasons why we frequently fail to meet expected standards. First, we must consider how common the condition is and resulting familiarity with it. Common conditions can lend themselves to efficient and protocolised treatment pathways.^16^ Seeing 45 cases over five years does not suggest this is a high-volume condition. On review with our team, this felt discordant and that several cases were missing from recent years. This may reflect issues with clinical coding, and lack of a standard radiological definition in reports.^13^ This may be of relevance to other services wishing to assess their treatment processes. Secondly, the admissions data show around one-third of patients (13/45) are direct admissions to general surgery, highlighting that the majority of cases are managed by other medical specialties, such as postoperative orthopaedic patients. All these teams will be able to perform basic assessment and treatment, but may lack the knowledge to facilitate additional treatments or when to refer, resulting in worse outcomes.^15,17^ Following this, if patients are managed remotely, there is a chance that they are lost to follow-up with continuity issues among on-call teams.

Compliance with the two guidance documents has not been reported in the literature, aside from an unpublished audit conducted in a cohort of 107 patients in Australia.^18^ This audit, similarly to our study, found that the SAGE guidelines are not adhered to routinely in practice in reference to the use of neostigmine as second-line treatment.^18^ In our study, neostigmine was used only in one patient in whom conservative management had failed. Endoscopic decompression seems to be used as the second-line treatment. This may be due to many reasons, including specific patient contraindications to neostigmine, clinician preference or unavailability of a monitored setting to administer neostigmine. Similarly to this audit, reasons for not using neostigmine were not documented.^18^ Another reason for this may be discrepancies in the current literature regarding the superiority of neostigmine over colonic decompression, with some studies reporting colonic decompression to be superior to neostigmine despite its recommendation as the second-line treatment.^10,19^ This is clearly an area for further investigation.

The main limitation of this study is that, due to its retrospective nature, it was heavily reliant on documentation in patient notes and various other electronic patient records such as electronic prescription charts. Upon review of records, documentation was missing in the paper notes but written in elsewhere on electronic notes or nursing care plans. It was also difficult to ascertain whether management had not been documented or actively not given and not documented. As a result, anything not documented was recorded as not present during data collection. Another limitation is the selection of patients for inclusion based upon radiologically confirmed ACPO. There was ambiguity in some CT scan reports that reported ‘ileus/pseudo-obstruction’ and it was difficult to ascertain whether a patient had radiologically defined ACPO, and thus these patients were excluded from inclusion. This is also reflected in the current published literature as no standardised definition of ACPO or diagnostic criteria exist, leading to inconsistency in research and reporting.^13^ Current guidelines recommend CT scanning for diagnosis of ACPO.^20^ Selecting patients on radiological diagnosis alone may have resulted in the exclusion of patients with ACPO who did not undergo a CT scan. Subsequently, this may have resulted in patients who met the inclusion criteria being discounted from the study population and thus the true number of patients with ACPO may not be accurate.

This study used a range of methods to identify potential patients, and undertook extensive and multiplatform notes review. This means the findings are as reliable as possible given the underlying dataset. Outcomes and treatment definitions were defined a priori, and with reference to published guidance.

Policy makers and clinicians should consider the findings of this study and relevance to their own practice. This is a low incidence, high morbidity condition that requires specialist input in a timely fashion. We recommend that local pathways and processes are implemented to ensure timely general surgery input, and access to endoscopic or pharmacological interventions to avoid the need for surgery. In units where guidelines and clinical pathways exist, we recommend addressing the barriers to guidelines' implementation related to that of individual practice, limited integration of departmental structures and organisational constraints.^21^

There is not a great deal of primary literature available to guide the management of ACPO. A PubMed search in July 2023 found 591 hits for the term ‘acute colonic pseudo-obstruction’. Of these, 95 were classed as reviews. Few randomised trials exist for the field to support these reviews. A meta-analysis of four randomised control trials, including 127 patients, found neostigmine to be effective in the management of ACPO following the failure of conservative management. However, each study varied in the number of participants, ranging from 21 to 42 patients, highlighting a small sample size and lack of literature on the topic.^22^ As this is a retrospective review of cases in a single institution, further research is required to determine the standard of care at multiple centres. Prospective data collection may also aid accurate reporting of care. We urgently need studies comparing timing of interventions, and comparison of pharmacological and endoscopic interventions. Further work to identify those at risk of developing ACPO would also help us to identify and treat patients early, as well as identify areas of care associated with poor outcomes.

## Conclusion

ACPO is an infrequent condition with high mortality. Adherence to published guidance is poor in the treatment of ACPO. This may be driven by frequent admissions to nongeneral surgical teams. Better data are needed to inform practice.
